# Reduced Physiological Complexity in Robust Elderly Adults with the APOE ε4 Allele

**DOI:** 10.1371/journal.pone.0007733

**Published:** 2009-11-05

**Authors:** Daniel Cheng, Shih-Jen Tsai, Chen-Jee Hong, Albert C. Yang

**Affiliations:** 1 Department of Microbiology, Immunology, and Molecular Genetics, University of California Los Angeles, Los Angeles, California, United States of America; 2 Department of Psychiatry, Taipei Veterans General Hospital, Taipei, Taiwan; 3 Divisions of Psychiatry, School of Medicine, National Yang-Ming University, Taipei, Taiwan; 4 Institute of Brain Science, National Yang-Ming University, Taipei, Taiwan; 5 Department of Psychiatry, Chu-Tung Veterans Hospital, Hsin-Chu County, Taiwan; 6 Institute of Clinical Medicine, National Yang-Ming University, Taipei, Taiwan; Institute of Preventive Medicine, Denmark

## Abstract

**Background:**

It is unclear whether the loss of physiological complexity during the aging process is due to genetic variations. The APOE gene has been studied extensively in regard to its relationship with aging-associated medical illness. We hypothesize that diminished physiological complexity, as measured by heart rate variability, is influenced by polymorphisms in the APOE allele among elderly individuals.

**Methodology/Principal Findings:**

A total of 102 robust, non-demented, elderly subjects with normal functions of daily activities participated in this study (97 males and 5 females, aged 79.2±4.4 years, range 72–92 years). Among these individuals, the following two APOE genotypes were represented: ε4 non-carriers (n = 87, 85.3%) and ε4 carriers (n = 15, 14.7%). Multi-scale entropy (MSE), an analysis used in quantifying complexity for nonlinear time series, was employed to analyze heart-rate dynamics. Reduced physiological complexity, as measured by MSE, was significantly associated with the presence of the APOE ε4 allele in healthy elderly subjects, as compared to APOE ε4 allele non-carriers (24.6±5.5 versus 28.9±5.2, F = 9.429, *p* = 0.003, respectively).

**Conclusions/Significance:**

This finding suggests a role for the APOE gene in the diminished physiological complexity seen in elderly populations.

## Introduction

Biologically, the physiological output of the human body emerges from interactions among a variety of factors, ranging from genes to organs to the environment [1]. These interactions, under healthy conditions, are essential for responses to environmental stress and are evident in both behavioral and physiological complexity, such as daily activities, heart rate, blood pressure, and brain electrical activities. In contrast, aging and illness are associated with degraded and/or decoupled regulatory networks and often result in the generation of less complex outputs [2], [3]. The loss of complexity is therefore, to a large extent, the hallmark of illness and the aging process [3], [4]. This reduced complexity can be quantified both behaviorally [4] and physiologically, such as through analysis of heart rate variability (HRV) [3]. However, despite a growing body of clinical and basic science research of applying complexity theory in aging and illness [2], [3], [4], [5], the relationship between the loss of such complexity and a genetic predisposition is still unclear.

The apolipoprotein-E (APOE) gene has been studied extensively in regard to its relationship to aging-associated medical illness, including cardiovascular disease [6], [7], [8], [9], geriatric cognitive decline [10], [11], and late-onset Alzheimer's disease [12]. The impact of the APOE polymorphism on the increased risk of a variety of medical illnesses might lead to a reduced life span and decreased adaptability of affected individuals to stress. These separate lines of evidence lead to our hypothesis that variants of the APOE gene (e.g., the ε4 allele) may potentially reduce physiological complexity in an affected individual, even before the onset of certain medical illness related to APOE variants. Therefore, in the present study, we applied a multiscale entropy (MSE) analysis to examine effects of the APOE genotypes on heart rate dynamics in a cohort of robust elderly adults.

## Results

**Figure 1:**
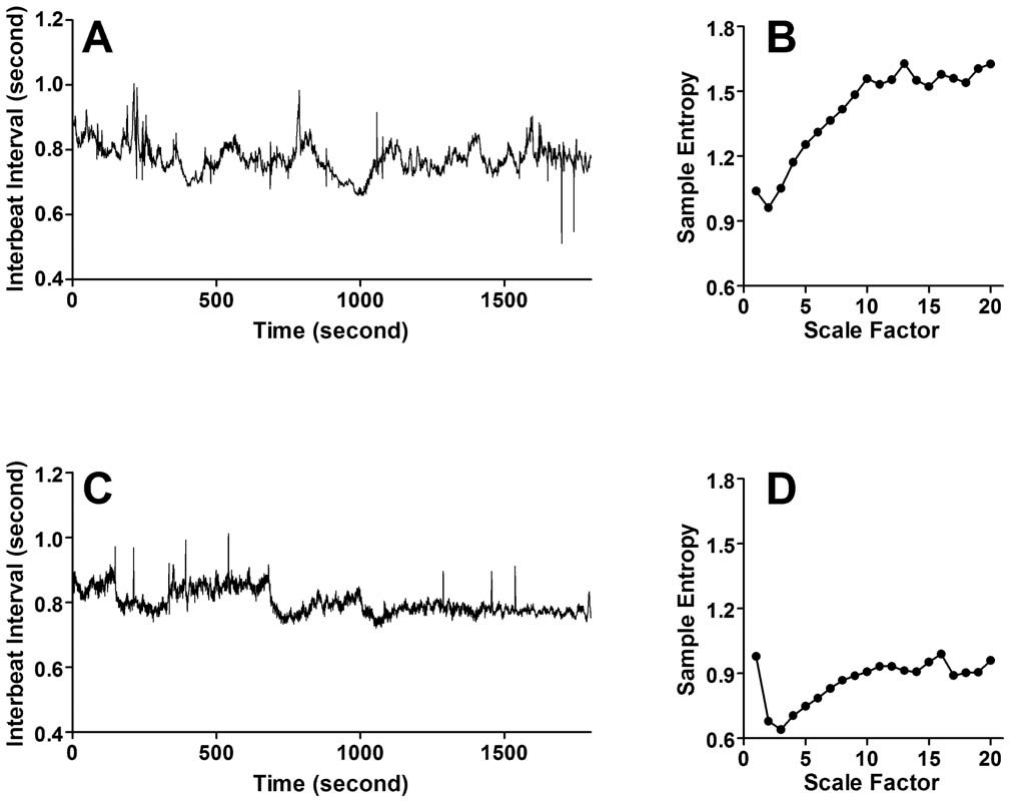
A comparison of a representative interbeat interval time series and analysis of multiscale entropy (MSE) between an APOE ε4-negative subject (top panels) and an APOE ε4-positive subject (bottom panels). Time series length is 30 minutes. The APOE ε4-negative subject showed multiscale organizations in fluctuations of interbeat intervals, whereas a relatively monotonic oscillation was seen in the interbeat interval time series obtained from an APOE ε4-positive subject. By considering the impact of scale on entropy calculations, the sample entropy values for the APOE ε4-negative subject is higher than that for the APOE ε4-positive subject for scales larger than two. Of note, the sum of MSE from scale factor 1 to 20 was 28.3 for the APOE ε4-negative subject and 17.3 for the APOE ε4-positive subject.

**Figure 2:**
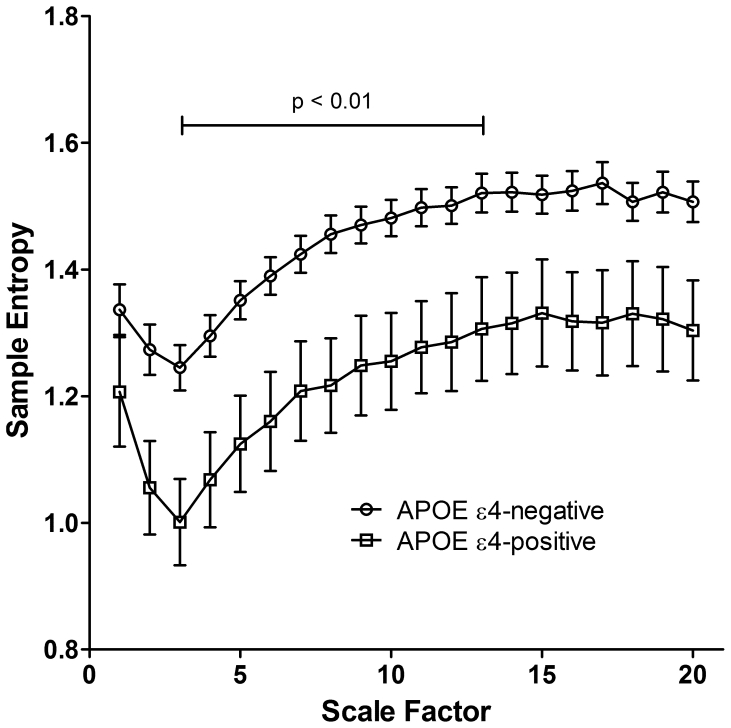
Multiscale entropy analysis by APOE ε4 genotype. Multiscale entropy was derived from two-hours of interbeat interval time series. Symbols represent mean values of entropy for each group and the bars represent the standard error. Parameters of sample entropy calculation are m = 2 and r = 0.15. The sample entropy values for subjects with APOE ε4 allele are significantly lower (*p*<0.01) on scales between 3 and 13, which are equal to oscillations at period around 10 to 40 heartbeats. *p* values were computed using Student's *t*-test at each scale factor.

Descriptive statistics are summarized in Table 1. Clinical characteristics were not different between the APOE ε4-negative and ε4-positive groups, except that the gender distribution was unbalanced between two groups (male, %: 97.7 vs. 80.0, *p* = 0.02, respectively). A comparison of representative interbeat interval time series and MSE analysis between an APOE ε4-negative and an APOE ε4-positive subject is shown in Figure 1. There were no significant differences in conventional HRV measures between two groups (Table 2). We also found no significant correlations between the MSE and conventional HRV indices. Significantly lower values of MSE were found in the ε4-positive group compared to ε4-negative group (24.6±5.5 vs. 28.9±5.2, F = 9.429, *p* = 0.003, respectively). No significant MSE by ANCOVA covarying for age or clinical parameters interaction effect was found. Figure 2 shows the comparison of the MSE analysis for the entire study cohort by the APOE ε4 genotype at different time scales. For scales ranging from 3 to 13 (equal to interbeat interval time series of 10 to 40 heartbeats), the sample entropy values were significantly lower (*t*-test, *p*<0.01) for the group of APOE ε4-positive, as compared to the group without the APOE ε4 allele.

**Table 1:** Demographics and clinical characteristics.

Characteristics	APOE ε4-negative N = 87	APOE ε4-positive N = 15	*t or χ^2^*	*p*
Age, year	79.1±4.4	79.4±4.2	−0.222	0.825
Male gender, n (%)	85 (97.7)	12 (80.0)	5.22	0.022
Education, year	7.2±4.3	7.1±5.5	0.106	0.916
Body mass index, kg/m^2^	23.8±3.1	24.8±3.0	−1.068	0.289
Hypertension, n (%)	44 (50.6)	9 (60.0)	0.16	0.689
Diabetes, n (%)	10 (11.5)	1 (6.7)	0.01	0.920
Stroke, n (%)	6 (6.9)	1 (6.7)	0.27	0.603
Current smoker, n (%)	22 (25.3)	2 (13.3)	0.46	0.498
Systolic blood pressure, mmHg	145±21	141±12	0.633	0.528
Diastolic blood pressure, mmHg	78±11	71±11	1.738	0.086
White blood cell count, 10^3^/mm^3^	7.3±2.9	7.5±2.3	−0.151	0.881
Hemoglobin, mg/dL	14.4±1.8	14.7±1.5	−0.591	0.556
Fasting glucose, mg/dL	89.7±15.8	89.0±13.1	0.136	0.892
Total cholesterol, mg/dL	194.7±48.6	182.2±19.3	0.839	0.404
Triglycerides, mg/dL	120.5±64.9	112.2±56.8	0.4031	0.688
Mini-mental state examination, score (0–30)	27.9±2.1	27.3±2.0	0.974	0.332

Values are mean ± standard deviation unless otherwise noted.

Categorical data are compared by chi-square tests, two tailed; all other *p* values are by Student's *t* test, two tailed.

**Table 2:** Heart-rate variability characteristics.

Variable	APOE ε4-negative N = 87	APOE ε4-positive N = 15	*F*	*p*
**Time Domain Measure**				
Mean heart rate, beat/min	77.9±16.0	80.9±16.6	0.454	0.502
Standard deviation of normal interbeat intervals, ms	70.3±32.4	72.1±27.8	0.027	0.871
Root mean square successive difference between adjacent normal interbeat intervals, ms	35.0±25.1	31.6±17.5	0.284	0.595
Percentage of adjacent normal interbeat intervals that varied by greater than 50 ms, %	12.7±18.0	8.0±8.8	0.991	0.322
**Frequency Domain Measure**				
Very-low-frequency power, ln(ms^2^/Hz)	7.94±0.88	7.88±0.93	0.079	0.779
Low-frequency power, ln(ms^2^/Hz)	6.41±1.07	6.21±0.94	0.491	0.485
High-frequency power, ln(ms^2^/Hz)	6.13±1.40	6.02±1.14	0.102	0.750
Low-frequency/high-frequency power, ln(ms^2^/Hz)	2.05±1.35	1.87±1.25	0.206	0.651
**Complexity Measure**				
Multiscale entropy, sum of sample entropy from scale factor 1 to 20	28.9±5.2	24.6±5.5	9.429	0.003

Values are mean ± standard deviation unless otherwise noted.

Power spectral estimates were log transformed due to skewed distributions. F ratios from analyses of covariance, controlling for age and clinical parameters.

## Discussion

Consistent with our hypothesis, the key finding of this study is that reduced physiological complexity, as measured by multi-scale entropy analysis, is associated with the APOE ε4 allele in this robust, aged population. The concept of loss of physiological complexity in illness and during the aging process has been hypothesized by several literature sources [3], [4], [13]. Degeneration of the control mechanisms by illness and aging may lead to a breakdown of coupling between physiological components and thus result in the loss of complexity in heart rate dynamics [3]. Moreover, the lack of associations between MSE and other time-frequency HRV measures indicates that MSE contains new information (complexity) which was not quantified by conventional HRV indices. The findings of a relationship between the APOE ε4 allele and reduced physiological complexity at different time scales suggest that variants of the APOE gene may affect the integrity of the physiological system during the aging process. While the exact mechanism of how the APOE ε4 allele affects physiological functions is unclear, degeneration of overall brain functions associated with the APOE ε4 allele [14], [15], [16], [17], which further results in decoupling between physiological control systems, is a possible mechanism underlying the association between APOE ε4 allele and reduced physiological complexity.

The present study employed a nonlinear method adapted from complexity theory, multi-scale entropy, to detect changes in physiological complexity in an aged population. Cardiovascular signals are largely analyzed using traditional time and frequency domain measures. However, such measures fail to account for important properties related to multi-scale organization and nonequilibrium dynamics [3]. The complementary role of complexity analysis is, therefore, an important tool to quantify the nonlinear properties of physiological signals. Of note, reduced complexity (e.g., fractal properties) have been implicated in the risk of fatal cardiac arrhythmia, increased mortality, or poor prognosis in cardiovascular diseases [18], [19], [20], [21], [22]. Our findings of a relationship between reduced physiological complexity and the APOE ε4 allele is in line with the observation that APOE ε4 increases the risk of cardiovascular events in the long run [7], [8]. Limited studies have shown procedures/exercise (e.g., meditation or Tai-Chi exercise) that are able to increase vagal tone could have protective effect on heart functions [23], [24]. However, it's not clear whether these preventive means also alter the physiological complexity. Therefore, further research is warranted to examine if an appropriate treatment/prevention could compensate the adverse impact of APOE ε4 allele on physiologic functions.

There are limitations to this study. First, an evaluation of cardiovascular function was not done and, thus, we cannot exclude the possibility that subjects with this genetic finding also had occult cardiovascular disease. Second, the possibility of selection bias cannot be excluded due to the relatively small sample of subjects and biased gender distribution towards the male gender (majority of study subjects were veterans). Prior reports have shown that gender may modulate the association between APOE gene and related neuropathology [9], [25], [26], and therefore may potentially affect the result of HRV analysis. Gender effect should be factored in the future study. Third, as the study design was cross-sectional, we cannot directly evaluate the long-term impact of the APOE polymorphism on the physiological complexity and incidence of cardiovascular diseases. A prospective study with a larger population should be done to address this issue. Finally, interbeat interval time series obtained from this aged population also posed a challenge to HRV analysis as ectopic heartbeats or cardiac arrhythmia (e.g., atrial fibrillation) are more common in elderly populations. However, the subjects in the present study were generally healthy and had no severe cardiac arrhythmia, thus permitting the feasibility of applying HRV analysis to these aged individuals.

In conclusion, reduced physiological complexity, as measured by complexity analysis, was significantly associated with the presence of the APOE ε4 allele in healthy elderly subjects. This observation may provide implications for understanding the role of genetic predisposition in physiological aging. Further large-scale research is warranted to investigate the interactions between genes, physiological functioning, and the environment in the elderly.

## Materials and Methods

### Ethics Statement

All subjects gave written informed consent before commencement of the study. The protocol was approved by the Institutional Review Board of the Taipei Veterans General Hospital.

### Study Sample

A total of 184 elderly Chinese-Han elderly volunteers were screened from a public veterans housing (N = 161) and a local elderly housing (N = 22) in the community. Of these subjects, 113 were successfully contacted for ambulatory electrocardiogram (ECG) monitoring. Each subject's history of medical disease, psychiatric illness, and medication use was evaluated carefully. Evaluation of the psychiatric history and of cognitive functioning was conducted by a psychiatrist. In addition, each subject underwent a basic physical examination and laboratory testing in order to gather data on clinical parameters. Subjects were screened and excluded if they had acute or major medical diseases (e.g., malignancy, heart failure, or infection), severe cardiac arrhythmia or frequent ectopic heartbeats, dementia (defined by clinical dementia rating scale >0.5), or a history of mental illness. Subjects were also excluded if they took medication with documented effects on the autonomic nervous system (e.g., beta-blockers). Of these 113 subjects, eleven were further excluded based on the above criteria. The final study sample consisted of 102 robust, non-demented elderly subjects with normal functions of daily activities (97 males and 5 females, aged 79.2±4.4 years, range 72–92 years).

### Laboratory Methods and ECG Monitoring

APOE genotyping was determined using PCR-RFLP, according to the procedure reported previously [27]. The studies of APOE and cognitive functions as well as heart rate variability and cognitive functions has been reported elsewhere [28], [29]. Of the 102 subjects, there were four genotypes: ε2/ε2 (n = 1, 1.0%), ε2/ε3 (n = 14, 13.7%), ε3/ε3 (n = 72, 70.6%), and ε3/ε4 (n = 15, 14.7%). When the sample was stratified according to the presence of the ε4 allele, 15 (14.7%) were ε4 carriers compared with 87 (85.3%) non-ε4 carriers. The frequency of ε4 allele was comparable with prior studies worldwide based on the community sample (7.9%–16.5%) [30], [31], [32], [33], [34], [35]. These subjects then underwent two-hour electrocardiogram (ECG) monitoring using a Holter monitor (MyECG E3-80 Portable Recorder, Microstar Inc., Taipei, Taiwan). All ECG monitoring took place in the daytime and participants were asked to stay in the resting state and to avoid smoking and drinking alcoholic beverages before the experimental procedures.

The Holter device continuously recorded three channels of ECG signals at a sampling rate of 250 Hz. The ECG signals were then processed and analyzed by an open source of HRV algorithms [36]. Briefly, after detecting the QRS complex on ECG by locating the R apex, the interbeat interval data was automatically calculated as the time interval between two consecutive R peaks (R-R interval). All beat annotations were carefully checked to avoid erroneous detections or missed beats.

### Analysis of Conventional Heart-Rate Variability

Conventional time and frequency domain HRV measures are employed. Time domain measures of HRV include the mean heart rate and standard deviation of the normal interbeat intervals (SDNN), the root mean square successive difference between adjacent normal interbeat intervals (RMSSD), and the percentage of adjacent intervals that varied by greater than 50 ms (pNN50) [37]. Spectral HRV measures [38] include high-frequency power (0.15–0.40 Hz), low-frequency power (0.04–0.15 Hz), and very-low-frequency power (0.003–0.04 Hz). Briefly, the RMSSD and pNN50 measure the short-term variation of interbeat intervals, which is mainly modulated by parasympathetic innervation [39]. Low-frequency power is suggested to be modulated by both sympathetic and parasympathetic activities, whereas high-frequency power is mainly modulated by parasympathetic activity [40], [41]. The low-frequency/high-frequency ratio was computed as a measure of the sympathovagal balance toward sympathetic activity [38], [42].

### Analysis of Physiological Complexity

Physiological signals under healthy conditions typically exhibit multi-scale variability, long-range correlations, and non-linearity [43]. Traditional complexity measurements are based on the concept of entropy which quantifes the regularity (orderliness) of a time series. Entropy increases with the degree of disorder and is maximum for completely random systems. However, an increase in entropy may not always be associated with an increase in dynamical complexity. For instance, a randomized surrogate time series has higher entropy than the original time series, despite the process of generating the surrogate data destroys correlations and degrades the information content of the original signal. A biologically meaningful complexity measure has been proposed by measuring the entropy over multiple time scales inherent in physiologic signals, termed multiscale entropy (MSE) [44]. MSE was computed over the interbeat interval data using publicly available algorithms from the PhysioNet [36], [44]. The algorithm of MSE is as follows: Given a one-dimensional discrete time series, {x_1_,…, x_i_;…,x_N_}, we construct consecutive coarse-grained time series, {y^(τ)^}, determined by the scale factor, τ, according to the equation: 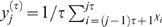, 1≤j≤N/τ. The length of each coarse-grained time series is equal to the length of the original time series divided by the scale factor, τ. For scale one, the time series is simply the original time series. We then calculate an entropy measure (sample entropy) for each coarse-grained time series and plot it as a function of the scale factorτ*τ*. The procedure and calculation of the MSE is summerized as following three steps: 1) construction of coarse-grained time series, 2) quantification of the sample entropy of each coarse-grained time series, and 3) summation of the sample entropy values over a range of scales. In the present study, sample entropy was calculated using a pattern length (m) of 2 and a similarity factor (r) of 0.15. The sum of sample entropy over all scale factors from 1 to 20 was computed for each subject and used to represent MSE in subsequent analyses. Of note, to reduce the non-stationarity beyond the maximum time scale detected by the MSE method (i.e., at scale 20, MSE can detect oscillation at the maximum of time period covering 61 heartbeats), interbeat interval time series was pre-detrended using the empirical mode decomposition method [45], [46].

### Statistical Analysis

We performed allele and genotype frequency and Hardy-Weinberg equilibrium tests for each APOE genotype. Chi-square tests were used to compare categorical variables. Student's *t*-test was used to test for differences in demographic and clinical measures between APOE ε4 carriers and non-carriers. Analysis of covariance (ANCOVA) was employed to determine group differences for the HRV variables, controlling for age and clinical parameters effects in this study sample. Partial correlation analysis was applied, controlling for age and clinical measures, to determine the associations between conventional HRV indices and the MSE measure. A more rigorous p-value of less than 0.01 (two-tailed) for the nature of association study was required for statistical significance. Based on prior literatures of applying MSE in healthy elderly subjects [44], [47], we estimated the total sample size to be at least 94 by assuming power of 80%, 1% significance level, minimum expected difference of 5 and estimated standard deviation of 5.
